# A randomised controlled trial exploring the impact of a dedicated health and social care professionals team in the emergency department on the quality, safety, clinical and cost-effectiveness of care for older adults: a study protocol

**DOI:** 10.1186/s13063-019-3697-5

**Published:** 2019-10-15

**Authors:** Marica Cassarino, Katie Robinson, Íde O’Shaughnessy, Eimear Smalle, Stephen White, Collette Devlin, Rosie Quinn, Dominic Trépel, Fiona Boland, Marie E. Ward, Rosa McNamara, Margaret O’Connor, Gerard McCarthy, Damien Ryan, Rose Galvin

**Affiliations:** 1 School of Allied Health, Faculty of Education and Health Sciences, Health Research Institute, Ageing Research Centre, University of Limerick, Limerick, Ireland; 2 Emergency Department, University Hospital Limerick, Limerick, Ireland; 3 Emergency Department, Our Lady of Lourdes Hospital, Drogheda, Ireland; 4 School of Medicine, Trinity College Dublin, Dublin, Ireland; 5 HRB Centre for Primary Care Research, Royal College of Surgeons in Ireland, Dublin, Ireland; 6 School of Psychology, Trinity College, The University of Dublin, Dublin, Ireland; 7 Emergency Department, St. Vincent University Hospital, Dublin, Ireland; 8 Graduate Entry Medical School, Faculty of Education and Health Sciences, University of Limerick, Limerick, Ireland; 9 Department of Ageing and Therapeutics, University Hospital Limerick, Limerick, Ireland; 10 Emergency Department, Cork University Hospital, Cork, Ireland; 11 Retrieval, Emergency and Disaster Medicine Research and Development Unit (REDSPoT), Emergency Department, University Hospital Limerick, Limerick, Ireland

**Keywords:** Emergency department, Health and social care professional, Team care, Older patients, Effectiveness

## Abstract

**Background:**

Older people are frequent emergency department (ED) users who present with complex issues that are linked to poorer health outcomes following the index visit, often have increased ED length of stay, and tend to have raised healthcare costs. Encouraging evidence suggests that ED teams involving health and social care professionals (HSCPs) can contribute to enhanced patient flow and an improved patient experience by improving care decision-making and thus promoting timely and effective care. However, the evidence supporting the impact of HSCP teams assessing and intervening with older adults in the ED is limited and identifies important methodological limitations, highlighting the need for more robust and comprehensive investigations of this model of care. This study aims to evaluate the impact of a dedicated ED-based HSCP team on the quality, safety, and clinical- and cost-effectiveness of care of older adults when compared with usual care.

**Methods:**

The study is a single-site randomised controlled trial whereby patients aged ≥65 years who present to the ED of a large Irish hospital will be randomised to the experimental group (ED-based HSCP assessment and intervention) or the control group (usual ED care). The recruitment target is 320 participants. The HSCP team will provide a comprehensive functional assessment as well as interventions to promote a safe discharge for the patient. The primary outcome is ED length of stay (from arrival to discharge). Secondary outcomes include: rates of hospital admissions from the ED, ED re-visits, unplanned hospital admissions and healthcare utilisation at 30 days, and 4 and 6 months of follow-up; patient functional status and quality of life (at baseline and follow-up); patient satisfaction; cost-effectiveness in terms of costs associated with ED-based HSCP compared with usual care; and perceptions on implementation by ED staff members.

**Discussion:**

This is the first randomised controlled trial testing the impact of HSCPs working in teams in the ED on the quality, safety, and clinical- and cost-effectiveness of care for older patients. The findings of this study will provide important information on the effectiveness of this model of care for future implementation.

**Trial registration:**

ClinicalTrials.gov, NCT03739515. Registered on 12 November 2018.

## Background

Internationally, emergency departments (EDs) face significant challenges in delivering high-quality and timely patient care set against a background of increasing patient numbers and limited hospital resources [1, 2]. An increasing ageing population and a higher number of individuals with multimorbidities are among the main demographic drivers of incremental ED attendances [3, 4], which in turn lead to ED crowding. Research has demonstrated that ED crowding contributes to a reduction in the quality of patient care, delays in commencement of treatment, increased length of the hospital admission, poorer adherence to recognised clinical guidelines, and increased overall costs [4, 5].

Evidence from international studies demonstrates that health and social care professionals (HSCPs) such as physiotherapists, occupational therapists and medical social workers can play a role in the ED in reducing length of patient stay, avoiding unnecessary hospital admissions and improving the patient experience [6–9]. Furthermore, promoting interdisciplinary care in the ED has been shown to enhance decision-making and contribute to timely and safe patient care, particularly for older adults [10–12]. A recent systematic review [6] demonstrated that care coordination teams comprising of HSCPs (including physiotherapists, occupational therapists and medical social workers) that provide early assessment and intervention to older adults in the ED can contribute to safer discharges and increased patient and staff satisfaction; however, the quality of the evidence is mixed, primarily due to inherent weaknesses in study designs and heterogeneity of patient groups and outcomes of interest.

The overall aim of this study is to examine the impact of a dedicated team of HSCPs in the ED on the quality, safety, and clinical- and cost-effectiveness of care of older adults in the ED.

The objectives of the study are as follows: 1) to implement an HSCP team including a whole time equivalent senior physiotherapist, senior occupational therapist and senior medical social worker in the ED at the University Hospital Limerick (UHL; Ireland) for a period of 6 months; 2) to examine if early assessment and intervention by the HSCP team improves the quality, safety, and clinical- and cost-effectiveness of care among older adults who present to the ED compared with usual care; and 3) to conduct a process evaluation for the HSCP intervention through focus group interviews with the HSCP team and representation from the wider ED staff regarding the implementation, delivery and acceptability of the intervention.

## Methods and design

### Design

**Fig. 1:**
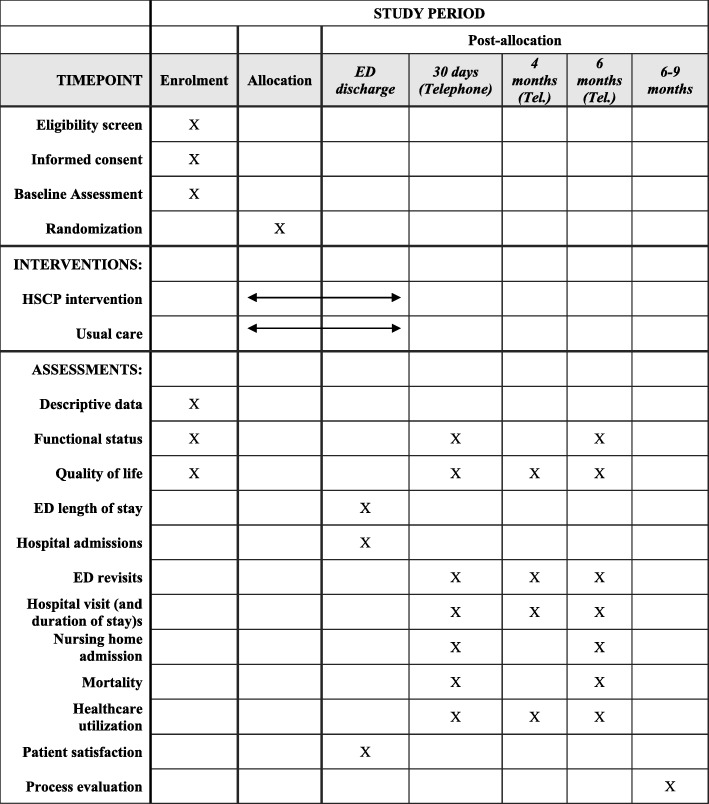
Study schedule. ED emergency department, HSCP health and social care professional

The study represents a single-centre parallel group randomised controlled trial which will compare assessment and/or interventions carried by an HSCP team, comprised of a senior physiotherapist, a senior occupational therapist and a senior medical social worker, in the ED with usual ED care. The Consolidated Standards of Reporting Trials (CONSORT) guidelines will be followed to ensure the standardised conduct and reporting of the research. This protocol has been registered on ClinicalTrials.gov (NCT03739515) and prepared in accordance with the Standard Protocol Items: Recommendations for Interventional Trials (SPIRIT) guidelines (see Fig. 1). The checklist is presented in Additional file 1.

### Setting

The study will take place in the ED of the UHL, a regional hospital with a large catchment area in the western region of the Republic of Ireland. Follow-up assessment will take place via telephone interviews.

### Participants

All adults aged ≥65 years who present to the ED at the UHL between December 2018 and May 2019 (inclusive) are considered eligible for inclusion to the study provided that they meet the following inclusion criteria: 1) the capacity (Mini-Mental State Examination ≥17) and willingness to provide informed consent; 2) baseline mobility and functional status; and 3) are medically stable (where relevant, see Table 1) and presenting with any of the complaints presented in Table 1 as per the Manchester Triage System 2–5 [13].

**Table 1:** Presenting complaint as per Manchester Triage System [13]

Before medical work-up*	After medical work-up**
Limb problems	Chest pain
Falls	Shortness of breath
Unwell adult	Abdominal pain
Back pain	Headache
Urinary problems	
Ear and facial problems	

*The health and social care professional (HSCP) team will proactively treat these individuals without prior assessment by a physician

**The HSCP team will await medical clearance prior to assessment and intervention

The exclusion criteria are: 1) aged under 65 years; 2) medically unstable; 3) neither the patient nor the carer can communicate in English sufficiently to complete consent or baseline assessment; and 4) presentation and discharge outside of HSCP operational hours (similar to other studies, the HSCP team is operational between the hours of 8 am and 5 pm Monday to Friday and, therefore, individuals who present to the ED and are discharged outside of these hours will not be included in the study).

This study is pragmatic in nature and, to reflect the realities of clinical practice in the ED, both the triage nurses and treating physicians will act as gatekeepers at the UHL site and inform eligible participants about the study. This method was chosen as the medical condition of participants will change over the course of the index admission.

### Consent

If participants and carers (where relevant) wish to hear more about the study, the triage nurse/treating physician will inform the dedicated research nurse or a member of the HSCP team who will provide the participant/carer with an information sheet and further discuss the nature of the project with them. Participants will be offered an opportunity to ask questions about participation in the study. Prospective participants will then be asked to sign a consent form. Participants will have the duration of their index admission to consider participation in the study. Consent and mechanisms relating to data control and processing will be compliant with the EU General Data Protection Regulation 2016/679 and also in compliance with the Data Protection Act 2018 (Section 36(2) Health Research Regulations 2018).

### Randomisation

Should participants explicitly consent to participate in the study, they will undergo a baseline assessment of function and quality of life by the research nurse or a member of the HSCP team. To minimise the possibility of selection bias, a researcher independent of the recruitment process (MC) will complete random group allocation. Computer-generated random numbers in blocks of 20 will be created using an internet-based system (https://www.randomizer.org/); the allocation equivalent to each number will be written in a sheet that will be placed in sealed opaque envelopes. These numbers will be stored in the pre-sealed envelopes in a locked drawer in the ED. Allocation will be revealed by the research nurse employed in the trial after recruitment of eligible participants and after conducting the baseline assessment. Allocation will be revealed by accessing and opening the next envelope in the sequence and providing the randomisation information simultaneously to the research team and patient. After allocation is revealed, participants will either receive the HSCP intervention or routine care (the control group).

### Power calculation

We estimated our sample size based on our primary outcome (ED length of stay) using G*Power version 3.1. Using data from the Patient Experience Time (PET) database employed in the ED at UHL, the average ED length of stay for patients aged 65 and older for the period 2016–2017 was 13.95 h (standard deviation 12.49 h) [14]. Estimating a 40% decrease in ED length of stay in the intervention group (mean 8.37 h), and with a 20% attrition rate to follow-up, a sample size of 258 patients (129 in each group) is required to achieve 90% power with two-tailed tests at an alpha level of 0.05.

### Experimental and control interventions

#### Intervention

Participants in the intervention group will be assessed by one or more members of the dedicated HSCP team (physiotherapy, occupational therapy and medical social work). This will include a holistic assessment of mobility, functional, cognitive and psychosocial abilities. Similarly, interventions prescribed by the HSCP team will be based on subjective and objective assessment of patients; individualised discharge care plans will be instituted from the ED to promote safe and supported discharge home. All assessments and interventions will be included in the medical chart of individual participants and communicated back to the ED team. To reduce the risk of contamination in the control group, the HSCP team’s activities will be limited only to the patients allocated to the intervention.

#### Control group

The comparison group will receive routine care for the duration of their stay in the ED. Currently, there is no dedicated team of HSCPs to assess and intervene with older adults who present to the ED at UHL. Ad-hoc services are provided by allied health professionals (i.e. physiotherapists or medical social workers not involved in the HSCP intervention for this study) if they are bleeped from their departments by the ED medical staff. This process will continue for the duration of the trial and will be recorded.

### Outcomes

A range of outcomes will be assessed to identify the potential impact of the intervention on quality, safety, and clinical- and cost-effectiveness of care. The primary outcome of the study is duration of patient ED stay (mean number of hours from time of arrival to discharge or admission). The secondary outcomes include the rates of hospital admissions from the ED (defined as the proportion of patients who are admitted to hospital after their index visits), the duration of hospital admission after the ED index visit, as well as the number of ED re-attendances, nursing home admissions, unplanned hospital visits (and duration of stay) and mortality within 30 days, 4 months and 6 months of the initial index visit. Healthcare utilisation (visits to a general practitioner (GP), public health nurse, home help, private consultation, outpatient department visit, or allied health services) will also be captured at 30 days, 4 months and 6 months after the index visit. Assessment of patient-oriented outcomes include the Barthel Index for Activities of Daily Living [15] as a global measure of function and the EuroQoL’s 5-level of the EQ-5D (EQ-5D-5 L) to measure health-related quality of life [16], which will be conducted at baseline as well as at follow-up (30 days and 6 months, with quality of life also assessed at 4 months).

In addition, patient satisfaction with their index visit will be explored using the 18-item Patient Satisfaction Questionnaire (PSQ-18) [17] at the time of the visit.

An economic analysis will estimate the incremental cost-effectiveness of the HSCP team from the perspective of the Irish public health service, compared with usual care. We will estimate healthcare costs from reference costs from national data sources. Participants’ responses to the EQ-5D-5 L questionnaire will be used to estimate health state utilities using the Irish value set [18], and quality-adjusted life years (QALYs) for each treatment group will be estimated across all time points.

Finally, a process evaluation will be conducted through a mixed quantitative–qualitative design to describe the implementation of the intervention as well as investigate the mechanisms and contextual influences of the implementation as perceived by the HSCP team and representation from the wider ED staff. A detailed study protocol for the process evaluation is available elsewhere [19].

### Data collection and management

Outcome assessment at baseline and at the end of the visit will be conducted by a research nurse blinded to the patient allocation in order to reduce potential detection bias. A chart review will take place by the research nurse to ascertain demographic details. Outcome assessment at follow-up (30 days, 4 months and 6 months following the index visit) will be conducted via a telephone call.

### Data analysis

Each participant in the study will be assigned a numerical code in order to link data collected at baseline to the data collected at the follow-up at 30 days, 4 and 6 months. Aggregate data will be anonymised. Appropriate descriptive statistics will be used to describe the baseline characteristics of study participants. These will include proportions, percentages, ranges, means and standard deviations, and medians and interquartile ranges (where data are not normally distributed). Differences between the two groups in terms of ED length of stay and hospital length of stay will be analysed using an independent samples *t* test if they meet the assumptions of normality; otherwise, we will employ the nonparametric alternative Wilcoxon Mann–Whitney test with bootstrapping to calculate an effect size 95% confidence interval (CI). The risk of hospital admission rates after the index visit, as well as ED re-visits, unplanned hospital admissions, nursing home admissions and healthcare utilisation at follow-up, will be estimated as odds ratios with 95% CI using a logistic regression, with analyses on follow-up measures adjusted for patient’s age and Identification of Seniors at Risk (ISAR) score at baseline. Patient functioning and quality of life at follow-up will be explored through an analysis of covariance (ANCOVA) adjusting for patient age, baseline ISAR score, baseline Barthel index (for function) and baseline quality of life (for quality of life). Differences in patient satisfaction with their index visit will be analysed using an independent samples *t* test with 95% CI.

For the cost-effectiveness analysis, and as per the Irish Health Information and Quality Authority (HIQA) guidance [20], the primary endpoint of the cost-effectiveness analysis will be costs, QALYs and the incremental cost-effectiveness ratio (ICER). Analysis of uncertainty of the joint distribution of cost and QALYs between the two arms of the study will be presented on a cost-effectiveness acceptability curve to indicate the probability that the HSCP intervention will be cost-effective, based on available trial data and across various willingness-to-pay thresholds.

### Monitoring

Participants will be under the medical care of their treating physician for the duration of their ED stay. Participants who are admitted to UHL as an inpatient will be transferred to a relevant ward following their ED stay where their medical care will be transferred to the relevant team. Participants who are discharged from the ED to the community setting or nursing home will be discharged to the care of their GP. The GP will be informed of their participation in the study. Participants may also be referred to community nursing, allied health professionals or community care teams. Once the study is completed, the health of participants will be monitored by their GP or treating physician (if the participant is an inpatient).

## Discussion

Based on the results of a systematic review [6], this is the first randomised controlled trial to examine the impact of an HSCP team on the quality, timeliness and cost-effectiveness of care of older adults in the ED when compared with usual care. Previous randomised controlled studies have focused mainly on single HSCPs working as members of ED teams [21, 22], while studies that have described HSCP teams have employed nonrandomised designs [23, 24]. Our study employs a controlled and robust design which is the most appropriate to demonstrate the efficacy of this model of care. The range of outcomes assessed in the study will enable us to provide detailed conclusions on the impact both at the patient and process levels. Furthermore, we will provide information on effectiveness both through a cost analysis and a qualitative investigation of feasibility involving ED staff members. The findings of the study will offer useful recommendations for future implementation.

A potential issue related to the study includes the fact that, due to the nature of the intervention, patients and ED staff members involved in the study cannot be blinded to allocation. While this may increase the risk of performance bias, an open procedure reflects realities of clinical practice in the ED. Another issue is linked to the working hours of HSCPs (8 am to 5 pm Monday to Friday) which may result in missing eligible patients who present out of these hours. However, we will capture crude estimates of these presentations to report the generalisability of the trial. Furthermore, as agreed with the team and the ED medical/nursing staff before the commencement of participant recruitment, the HSCP team’s scope will be limited only to patients involved in the trial; however, a risk of contamination cannot entirely be ruled out as ED medical and nursing staff collaborating with the HSCP team could be influenced in their procedures if taking care of patients in the control group.

## Trial status

This is Protocol version 1. At the time of the manuscript submission (June 2019), the status of the trial is ‘Recruitment completed’. Participant recruitment began on 3 December 2018 and was completed on 31 May 2019 (inclusive). Follow-up data collection is estimated to be completed by November 2019 (inclusive).

## Supplementary information

**Additional file 1.** SPIRIT 2013 Checklist: Recommended items to address in a clinical trial protocol and related documents.

## Data Availability

Data sharing is not applicable to this article as no datasets were generated or analysed during the current study.

## Abbreviations

CI: Confidence interval
CONSORT: Consolidated Standards of Reporting Trials
ED: Emergency department
EQ-5D-5 L: 5-level EuroQoL 5D version
GP: General practitioner
HIQA: Health Information and Quality Authority
HSCP: Health and social care professional
ICER: Incremental cost-effectiveness ratio
ISAR: Identification of Seniors at Risk
QALY: Quality-adjusted life year
SPIRIT: Standard Protocol Items: Recommendations for Interventional Trials
